# *Mycoplasma pneumoniae* Infection in Children Is a Risk Factor for Developing Allergic Diseases

**DOI:** 10.1155/2014/986527

**Published:** 2014-04-07

**Authors:** Qing Ye, Xiao-Jun Xu, Wen-Xia Shao, Yan-Xiang Pan, Xue-Jun Chen

**Affiliations:** ^1^Clinical Laboratory, The Children's Hospital, School of Medicine, Zhejiang University, Hangzhou 310003, China; ^2^The Department of Hematology/Oncology, The Children's Hospital of Zhejiang University School of Medicine, Hangzhou 310003, China; ^3^Clinical Laboratory, Hangzhou First People's Hospital, Hangzhou 310003, China

## Abstract

*Mycoplasma pneumoniae *(MP) infection is the dominant cause of pneumonia in children. We sought to determine the relationship between MP infection and secondary allergic disease and to clarify the associated mechanisms of inflammatory response. A prospective study was performed among 1330 patients diagnosed with pneumonia to investigate the patient immune status by determining the correlation between MP infection, immunoglobulin E (IgE) levels, and a spectrum of associated serum cytokines. Serum IgE, IL-4, IL-6, and IL-10 levels for MPP patients in the acute phase were obviously higher than those in the recovery phase (*P* < 0.01). MPP patients with allergic conditions had increased serum IgE levels and increased IL-4/INF-**γ** ratio, and IgE and Eosinophil Cationic Protein were further elevated in patients who eventually developed secondary asthma changes. Patients with severe pneumonia and high clinical pulmonary infection scores presented higher levels of IL-4 and IL-5 in serum than those with low scores (*P* < 0.01). The proportion of CD4+ and CD8+ T cells that secreted IL-4 was significantly increased in MPP patients with elevated IgE. Our data demonstrate a significant correlation between MP infection and IgE levels, which is associated with a Th1/Th2 cytokine imbalance.

## 1. Introduction

*Mycoplasma pneumoniae* (MP) is an extracellular pathogen that attaches to ciliated epithelial cells of the respiratory tract mucosa and causes damage [1, 2]. MP infection is the dominant cause of pneumonia in children [3–6], who present with more than 40% of community-acquired pneumonia [7–9]. Children aged 4-5 years are most likely to suffer from MP infection, with an epidemic cycle of 3-4 years [9].

In the clinic, patients with MP infection are more likely to develop symptoms such as paroxysmal and successional coughing and wheezing, often accompanied by type 1 hypersensitivity disorders such as allergic rhinitis or eczema [10, 11]. These are also our unpublished observations. Elevated immunoglobulin E (IgE) levels are often observed in the serum of patients with MP infection in our clinical practice. In order to clarify the mechanism of MP infection-related type 1 hypersensitivity disease for children with pneumonia, we launched a prospective study to determine the spectrum of serum cytokines and a series of other immune indices, including levels of IgE, IL-4, IL-6, IL10, and INF-*γ* and T-cell status in children with pneumonia at acute and convalescence phases of MP infection. The relationship between the above parameters and disease severity was analyzed to establish a mechanism of pathogenesis that contributes to allergic diseases following MP infection.

## 2. Materials and Methods 

### 2.1. Diagnosis of MP Pneumonia (MPP)

This study was performed prospectively from January 2011 to October 2012 and was approved by the Institutional Review Board of Zhejiang University. Informed written consent was obtained from guardians on the behalf of the minor/child participants involved in the study. Patients with preexisting allergic disease, with the exception of drug allergies, were excluded from the study. Pneumonia was diagnosed on the basis of radiological findings and clinical features, including paroxysmal cough, expiratory dyspnea, fever, and rale. MP infection was evaluated by ELISA to determine antimycoplasma antibody titers and/or PCR to detect MP DNA in nasopharyngeal swabs. Positive MP-IgM and/or MP-IgG antibody titers that were more than fourfold higher in the recovery phase than in the acute phase were regarded as positive results [12–14]. For the PCR analyses, MP genes encoding the 16S ribosomal RNA and the P1 adhesion protein were amplified with multiple primer sets. All targeted gene sequences were MP specific [15, 16]. The presence of other pathogens was tested in parallel using specific laboratory tests, including immunofluorescence to detect respiratory virus including adenovirus, respiratory syncytial virus, parainfluenza virus, influenza virus, and EB virus; sputum and blood cultures for bacteria; and PCR to detect* Chlamydia pneumoniae*. If any of these assays for other pathogens tested positive, the patient was excluded from the study.

### 2.2. Patient Definitions

The acute phase was defined as typical clinical manifestations of MP pneumonia; MP DNA and/or MP IgM test were positive. The recovery phase was defined as at least one week after clinical symptoms improved with normal levels of white blood cells, C-reactive protein, and biochemical indexes; MP DNA test was negative; MP IgG test was positive.

MPP patients were defined as an allergic condition: they were allergic to more than two allergens as determined by specific IgE as well as total IgE to a standard panel of allergens.

Critically ill MPP cases were divided into two groups according to the clinical pulmonary infection score (CPIS), as defined in 2001 by the American Thoracic Society: scores of 6 points or higher were defined as high CPIS, while scores lower than 6 points were defined as low CPIS [17]. Fifty healthy children who came to the clinic for routine physical examination were randomly selected as the control group.

### 2.3. Measurement of Serum Cytokines, IgE, and ECP

Quantitative analyses of INF-*γ*, IL-4, IL-5, IL-6, and IL-10 levels in serum were achieved simultaneously after blood clotting using CBA Human Cytokine kits (BD Biosciences, San Jose, CA, USA) as described [18]. Samples were quantitatively analyzed with an established standard calibration curve for each kit.

Serum total IgE and Eosinophil Cationic Protein (ECP) levels were determined using UniCAP total IgE and ECP kits (Pharmacia), as previously described [19].

### 2.4. Flow Cytometry for Cell Surface Molecules and Intracellular IL-4 Staining

To quantify the proportion of CD4+/CD8+ cells secreting IL-4, specimens were processed by cell surface staining and FACS as described [20, 21]. In brief, cells were washed and resuspended at 107 cells/mL in Fc blocker (BD Pharmingen). Then (0.5–1) × 106 cells were stained using specific fluorochrome-conjugated antibodies against CD3, CD4, and CD8 antigens (BD Pharmingen) for 30 min at 4°C. An equal volume of formalin was mixed with the cells to fix them, and then these cells were washed, resuspended in FA buffer, and analyzed by FACS.

FACS analysis of intracellular IL-4 production was performed in combination with CD4+ and CD8+ cell surface marker expression in peripheral blood lymphocytes. Specimens were preprocessed as described in the literature [22]. To increase the content of intracellular cytokines before staining, cells were then stimulated for 5 h* in vitro* with ionomycin (1 *μ*g/mL) and phorbol myristate acetate (50 ng/mL) prior to exposure to brefeldin A. The surface molecules were stained using specific antibodies, and then the cells were washed two times. Intracellular IL-4 was stained using a BD Cytofix/Cytoperm kit according to BD Pharmingen's instructions. Test results were collected on a FACSCalibur flow cytometer (BD Pharmingen) and analyzed with FlowJo software (Tree Star).

### 2.5. Statistical Analysis

The test results are presented as means ± standard deviation (SD). Comparisons between two groups were performed by two-tailed independent-sample *t*-tests, while comparisons among three or more groups were performed by one-way analysis of variance (ANOVA) with a Bonferroni multiple comparison post hoc test. Results were considered statistically significant for *P* value less than 0.05. All statistical analyses were performed by using SPSS 18.0 software.

## 3. Results

### 3.1. Patients Characteristics

**Figure 1:**
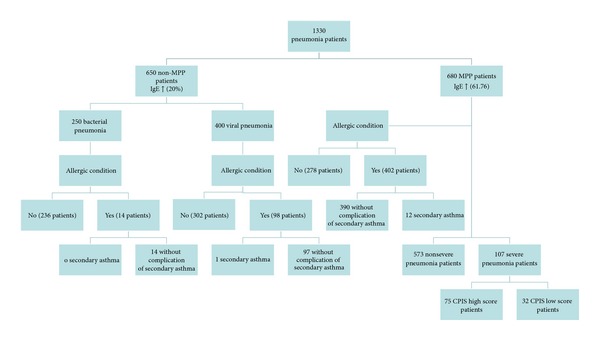
Characterization of pneumonia patients. The flow chart shows the categorization of the 1330 patients in this study. The 650 pneumonia patients without MP infection (non-MPP patients) tested positive for either bacterial (*n* = 250) or viral (*n* = 400) infection, and 20% had elevated IgE levels (>100 IU/mL). Further categorization is shown for the presence of allergic conditions. Of the 680 pneumonia patients with MP infection (MPP patients), 61.76% had elevated IgE. The MPP patients could be further subdivided by the occurrence of allergic conditions (*n* = 278 without and *n* = 402 with allergic conditions), including those with allergic conditions resulting in secondary asthma (*n* = 390 without and *n* = 12 with secondary asthma) or they could be subdivided by the severity of the pneumonia (*n* = 573 with nonsevere pneumonia and *n* = 107 with severe pneumonia), with the severe pneumonia categorized with high or low clinical pulmonary infection score (CPIS) (75 or 32 patients, resp.).

This study was performed prospectively from January 2011 to October 2012. The research involved 1330 children who were admitted to the pneumology department with pneumonia, including 650 patients without MP infection (non-MPP patients) and 680 patients with MP infection (MPP patients; Figure 1). Patients with MPP were characterized by the occurrence of allergic conditions (*n* = 402), including the development of secondary asthma (*n* = 12), and by the occurrence of severe pneumonia (*n* = 107). The patients with severe pneumonia were further subdivided by CPIS ≥ 6 (*n* = 75) or CPIS < 6 (*n* = 32). The average age of the patients with MPP was 3.2 ± 3.6 years (range, 0.08–16 years), while the average age of the non-MPP patients with pneumonia was 3.5 ± 3.8 years (range, 0.05–15.8 years). Fifty control subjects without pneumonia were also compared in this study.

### 3.2. Correlation of MP Infection with Allergic Conditions and IgE Levels

Analysis of the 1330 patients with pneumonia in Figure 1 suggests that 59.1% (402/680) of the MPP patients had concomitant allergic conditions, whereas only 5.6% (14/250) of the non-MPP patients with bacterial pneumonia and 24.5% (98/400) of the non-MPP patients with viral pneumonia had allergic conditions. To determine whether these trends are associated with similar variability in IgE levels, we collected serum for the 1330 patients. A similar correlation was found between MP infection and IgE levels. Among the 680 MPP patients, 61.76% had elevated IgE levels (>100 IU/mL), while among the 650 non-MPP pneumonia patients, only 20.0% had elevated IgE levels (*P* < 0.01) (Figure 1). Because IgE levels are associated with type I hypersensitivity reactions, these results suggest that MP-infected patients have an increased risk of developing allergies and/or asthma that is reflected by the serum IgE content.

### 3.3. Levels of IgE, Cytokines, and T-Cell Surface Markers during the Acute and Recovery Periods of MPP

To further determine whether IgE levels vary according to the progression of MPP, and to determine whether additional immunological markers also may be modulated over the course of this infection, we measured the serum levels of IgE, IL-4, IL-6, IL-10, and INF-*γ* and the percentages of CD3+, CD4+, and CD8+ cells in the acute and recovery phases of MP pneumonia as compared to non-MPP patients and normal controls (Table 1). Levels of IgE, IL-4, IL-6, and IL-10 were significantly higher in serum from acute MPP patients than in serum from normal controls (*P* < 0.01), suggesting the occurrence of a Th2-type response. Furthermore, levels of these immune mediators in MPP patients at the acute phase were significantly higher than those at the recovery phase (*P* < 0.01). However, the INF-*γ* levels and percentages of CD3+, CD4+, and CD8+ cells were comparable for patients at each phase of MPP and for normal controls (*P* > 0.05). These results are consistent with the association of a Th2 response in MPP that is more prominent in the acute phase, but they also suggest that interleukin production may not be associated with imbalance of CD4+/CD8+ cell.

### 3.4. The IgE and IL-4 versus INF-*γ* Levels in MPP Patients with or without Allergic Conditions

**Figure 2:**
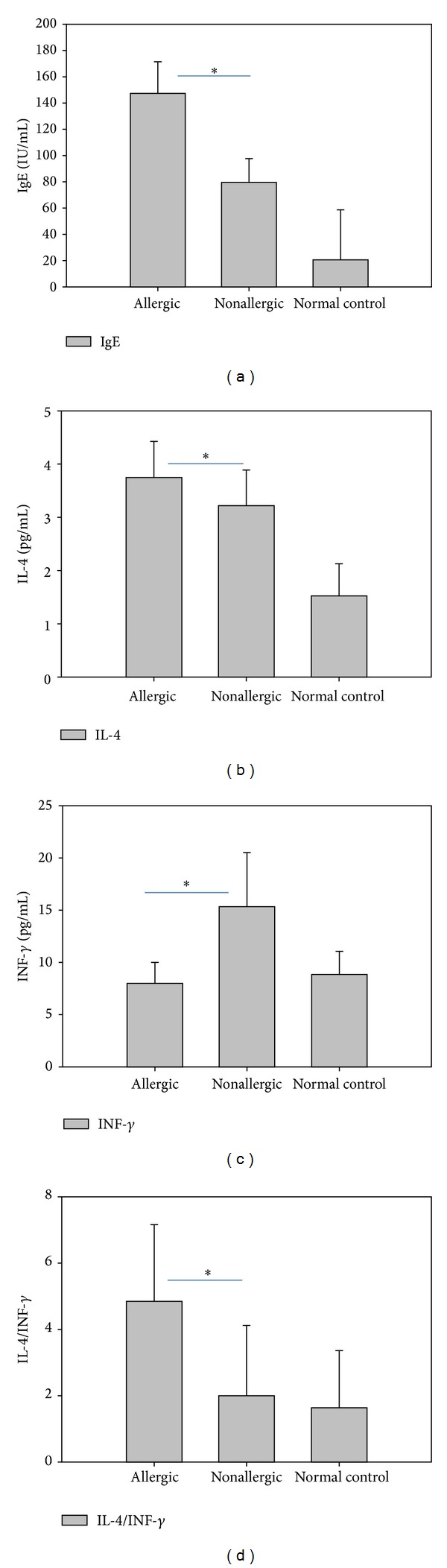
The IgE and serum cytokine levels for MPP patients with or without allergic conditions. Levels of IgE and serum cytokines were measured for *n* = 402 MPP patients with concomitant allergic conditions and *n* = 278 MPP patients without allergic conditions. The results for *n* = 50 control subjects without pneumonia are shown for comparison. Results represent the mean ± standard deviation. *P < 0.01 for allergic versus nonallergic patients.

To further determine the association of serum levels of IgE, IL-4, and INF-*γ* with allergic conditions among the MPP patients, we calculated the mean expression of each of these proteins for the 402 patients with and the 278 patients without allergic conditions. All “allergic conditions” happened after MPP among patients without preexisting allergic disease/asthma. As shown in Figure 2, the IgE and IL-4 concentrations in the serum of MPP patients with allergic conditions were significantly higher than those without allergic conditions (*P* < 0.01). Conversely, the INF-*γ* was significantly lower (*P* < 0.01) in allergic MPP patients, with a corresponding increase in the IL-4/INF-*γ* ratio. These results verify the association of a Th2-type allergic response that involves increased production of IgE and increased IL-4 versus INF-*γ* in MPP patients with allergic conditions.

### 3.5. Association of ECP and IgE Levels with the Development of Secondary Asthma

**Figure 3:**
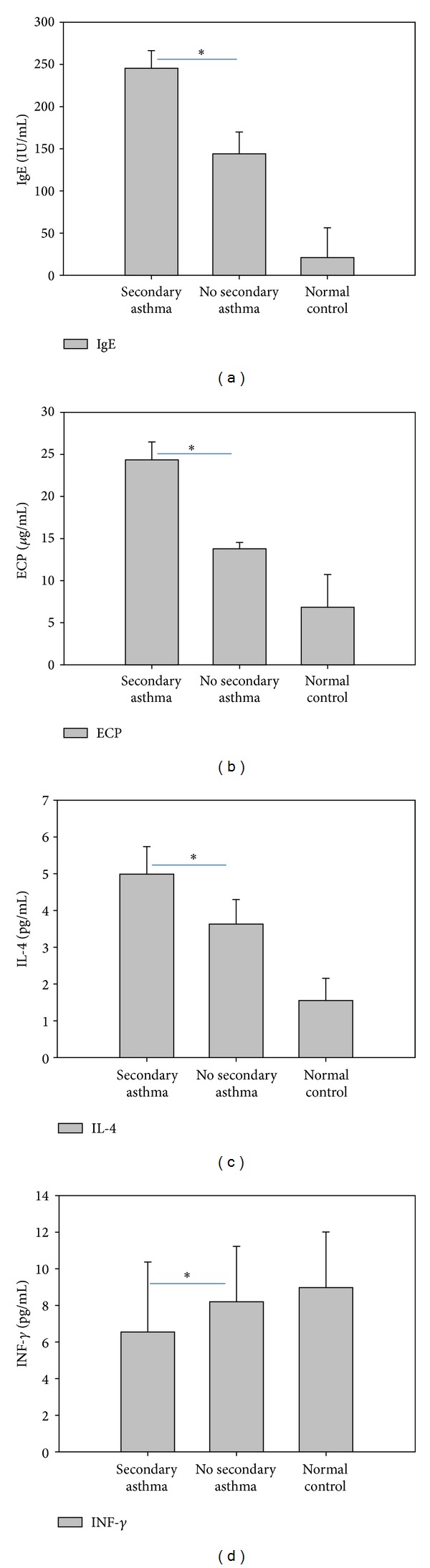
The IgE, ECP, and serum cytokine levels for MPP patients with allergic conditions, with or without secondary asthma. Levels of IgE, ECP, IL-4, and INF-*γ* were measured for MPP patients with allergic conditions who developed secondary asthma (*n* = 12) or did not develop secondary asthma (*n* = 390). The results for *n* = 50 control subjects without pneumonia are shown for comparison. Results represent the mean ± standard deviation. *P < 0.01 for secondary asthma versus no secondary asthma.

Next, we determined whether the differing allergic response can be observed for MPP patients that develop secondary asthma. Among the 402 MPP patients with allergic conditions, the 12 patients (3.0%) who developed secondary asthma had ECP and IgE levels of (24.31 ± 2.12) *μ*g/L and (245.25 ± 20.1) IU/mL, respectively. These concentrations were much greater than those for the remaining 390 MPP patients without complications from secondary asthma (IgE, 13.76 ± 0.75 *μ*g/L, *P* < 0.01; ECP, 143.5 ± 25.35 IU/mL, *P* < 0.01) (Figure 3). These results suggest a role for ECP and IgE in the development of secondary asthma.

### 3.6. Serum Cytokines in High and Low CPIS Score Groups

**Figure 4:**
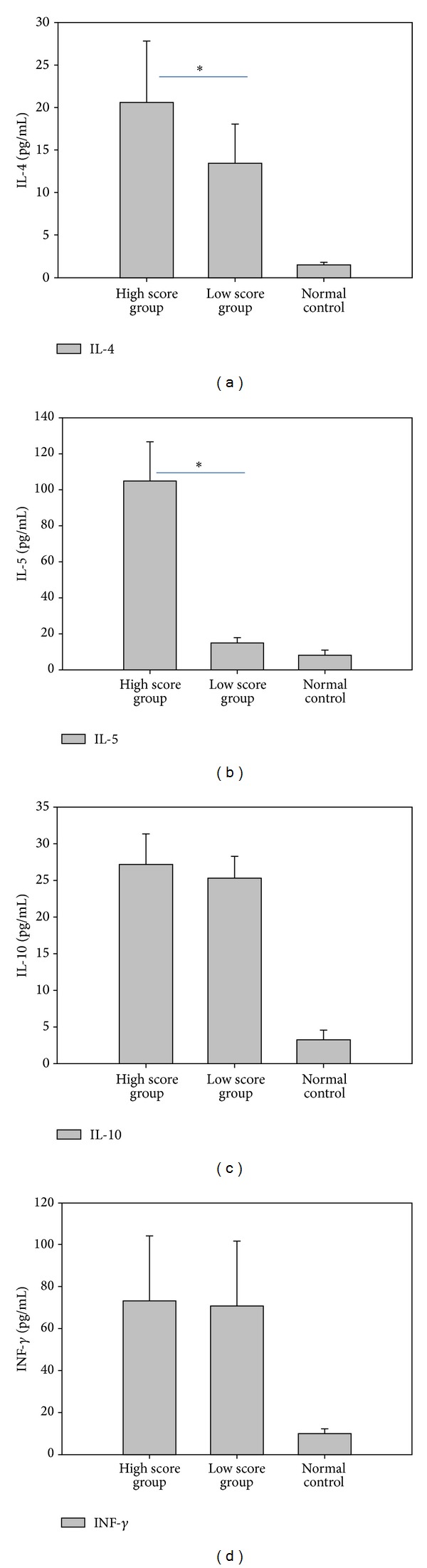
Serum cytokine levels in MPP patients with severe pneumonia and high or low CPIS values. Among the 107 patients with severe MPP, serum cytokine levels were assessed in those with high CPIS (≥6 points; *n* = 75) and low CPIS (<6 points; *n* = 32). The results for *n* = 50 control subjects without pneumonia are shown for comparison. For all four cytokines tested, the levels for both the high and low score groups were statistically higher than the levels in the normal control subjects, while the IL-4 and IL-5 levels were further elevated in the high score group. Results represent the mean ± standard deviation. *P < 0.01 for high versus low CPIS groups.

To examine the trend of increased cytokine levels under extreme conditions of the disease, we determine the levels of cytokines among 107 MPP patients that were classified as having severe pneumonia, including 75 with high CPIS values and 32 with low CPIS values. The levels of serum IL-4, IL-5, IL-10, and INF-*γ* in both the high and low CPIS groups were higher than those in normal control (*P* < 0.01). Furthermore, IL-4 and IL-5 levels in the high CPIS score group were statistically higher than in the low CPIS group (*P* < 0.01) (Figure 4). These results suggest that serum IL-4, IL-5, IL-10, and INF-*γ* levels may correlate with the severity of pneumonia, and that the most severe cases as assessed by CPIS are characterized by more highly elevated IL-4 and IL-5 levels.

### 3.7. IL-4 Production in T Cells from MPP Patients with Differing IgE Levels

**Figure 5:**
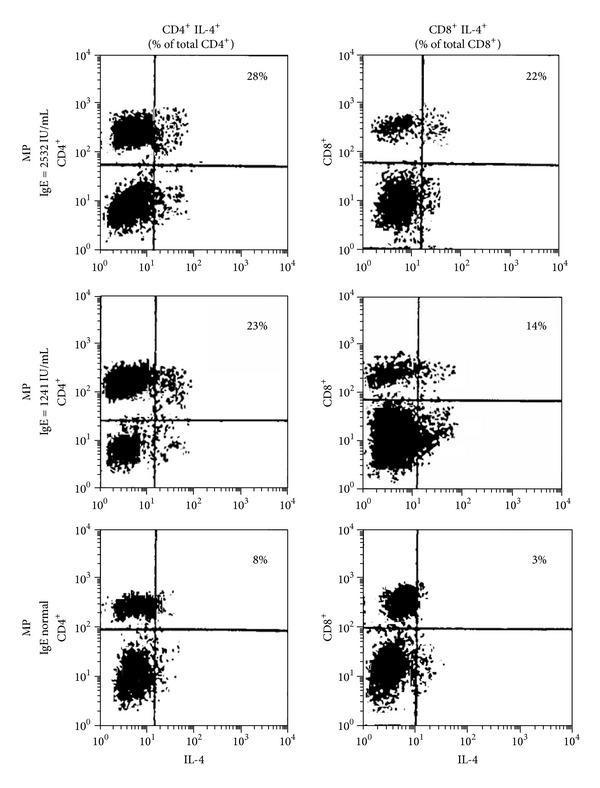
Representative FACS analysis of intracellular IL-4 and either CD4 or CD8 cell surface marker expression in peripheral blood lymphocytes from patients with normal or elevated IgE levels. FACS analysis is shown for a patient with normal IgE levels, a patient with moderately elevated IgE levels (1241 IU/mL), and a patient with severely elevated IgE levels (2532 IU/mL). Results shown are representative of *n* = 40 MPP patients with elevated IgE and *n* = 20 MPP patients with normal level IgE. The proportion of cells secreting IL-4 is indicated for the CD4+ and CD8+ cells of each group.

Our results thus far indicate that MPP patients have higher levels of serum IgE and IL-4, which suggests an increased Th2 response (Table 1 and Figures 2–4). To verify these findings at the cellular level, we performed intracellular staining combined with cell surface marker analysis for CD4+ and CD8+ T cells. A sharp increase in IL-4 after PHA stimulation was observed for both CD4+ and CD8+ T-cell subsets for MPP patients with elevated IgE. Furthermore, the elevation in IL-4 levels was proportional to the elevation in IgE levels (Figure 5). These results confirm that increased IL-4 levels correlate with IgE production.

## 4. Discussion 

Excessive production of IgE plays a major role in the pathogenesis of allergic diseases such as drug allergy and other type I allergies [23–26]. Clinical symptoms of type I allergic responses are due to a small group of plasmacytes that secrete IgE antibodies, which bind to high-affinity Fc*ε*RI receptors on basophils and mast cells, and these cells become sensitized. When the sensitized cells are stimulated by allergens, the IgEs on basophils and mast cells are rapidly crosslinked. This crosslinking triggers the release of large amounts of histamine, as well as other mediators of inflammation [27]. Patients with MP infection are more likely to develop type 1 hypersensitivity disorders such as allergic rhinitis or eczema [10, 11], and we therefore performed a comprehensive analysis to determine the association of IgE levels with the allergic symptoms of MPP. We found that IgE is significantly increased in MPP patients, especially in the acute phase and in patients with allergic status, indicating that MP may mediate a type I hypersensitivity disorder during the development of MPP.

Investigation of factors that influence IgE production is important for understanding the cellular mechanisms that lead to increased IgE in MPP patients. It is well established that the signal delivered by IL-4 is a crucial event of class switching to IgE. IL-4 regulates the induction of germ-line *ε* transcripts during the critical molecular event of antibody-type conversion [28–30]. Furthermore, IL-4 produced by Th2 cells can block cytokine production by Th1 cells, and conversely, INF-*γ* produced by Th1 cells is known to downregulate Th2 immune response, inhibiting anaphylaxis [31, 32]. This study shows that IL-4 is increased, while INF-*γ* is decreased in MPP patients, thus providing a molecular basis for the increased IgE levels and suggesting that a Th1/Th2 cytokine imbalance occurs in the process of MP infection. IL-4, IL-6, and IL-10 levels in the acute stage of MPP were higher than those in the recovery period. Additionally, for the most severe patients, the IL-4 and IL-5 levels paralleled the CPIS score and reflected the severity of MP pneumonia. In contrast, the IL-10 level in the CPIS high group was just slightly higher than in the CPIS low group, suggesting that IL-10 does not reflect the severity of MPP. This may be because of the basic function of IL-10 in preventing Th1 cells from producing cytokines as opposed to the Th2 function of the other cytokines.

Our results also suggest that the Th1/Th2 cytokine balance may impact the future outcome of MPP. For the 12 cases of MPP in which the children developed secondary asthma, we found that the ECP and IgE levels were key factors. The ECP and IgE levels of MP pneumonia patients who developed secondary asthma were all significantly higher than those without secondary asthma (*P* < 0.01). The elevated ECP levels may be associated with damage to the respiratory epithelium and accelerated hypersensitivity in the respiratory system [33].

Despite the clear association with a Th2 pathway, we did not observe statistical differences between the CD4+ and CD8+ T-cell numbers in the acute and recovery phases for patients with MPP. The body's immune balance depends on mutual coordination and control of lymphocyte subsets, especially for CD4+ and CD8+ T cells, which engage in a T-cell network by maintaining their own stability to adjust the immune response [34, 35]. In animal experiments, CD8+ T cells are able to regulate IgE responses, although IgE response is affected by the timing of CD8+ cell depletion, suggesting that a complex mechanism exists. In order to clarify the role of CD4+ and CD8+ T cells in the process of allergic disease caused by MP infection, we used intracellular cytokine staining combined with cell surface marker analysis to reveal if an increase in IL-4 production for MPP was associated with elevated IgE. The results show that a sharp increase of IL-4 production in both CD4+ and CD8+ T cells occurs in MPP patients with elevated IgE after PHA stimulation. The IL-4 production level rose incrementally with elevated IgE for both CD4+ and CD8+ T cells. Thus, these results support a mechanism whereby elevated IL-4 in the acute period of MP infection promotes the proliferation and differentiation of Th2-like CD8+ T cells, and then these Th2-like CD8+ T cells secrete more IL-4 to enhance the function of CD4+ Th2 T cells, which in turn secrete IL-4 to accelerate the switch of immature B cells to IgE. Future studies to characterize different Th1 and Th2 CD8+ T-cell clones and to determine their role in class switching of IgE will help to clarify this mechanism.

## 5. Conclusion

**Figure 6:**
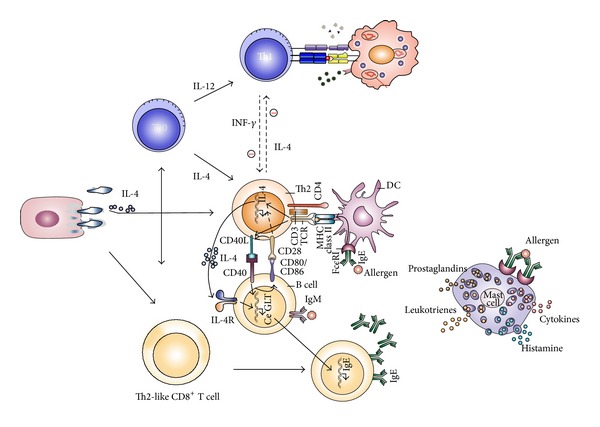
Proposed role of* Mycoplasma pneumoniae* in type I hypersensitivity. The adherence of MP to respiratory ciliated epithelium causes the production of cytokines and recruitment of lymphocytes and other immune cells. These cytokines influence the Th1/Th2 balance, which leads to the elevated secretion of IL-4 by Th2 cells. The production of IgE antibodies is enhanced by IL-4 receptor signals. Subsequent crosslinking of IgE on basophils and mast cells triggers their degranulation and the release of active substances.

In short, our results support a model (Figure 6) in which the adherence of MP to respiratory ciliated epithelium cells causes the production of cytokines and recruitment of lymphocytes and other immune cells. These cytokines influence the balance of Th1/Th2 T cells, leading to a predominance of Th2-like CD4+ and CD8+ T cells, which secrete more IL-4. The production of IgE antibodies is activated by signals that are amplified by the IL-4 receptor. Allergen-specific IgE binds to high-affinity Fc*ε*RI receptors on basophils and mast cells. After sensitization by antigen, exposure to the allergen induces IgE crosslinking, which triggers the degranulation and release of active substances including enzymes, histamine, and cytokines that mediate the clinical manifestations of atopy.

## Figures and Tables

**Table 1:** The levels of IgE, cytokines, and T-cell CD positivity in the acute and recovery phases of *Mycoplasma pneumoniae* pneumonia (MPP), bacterial pneumonia, and viral pneumonia.

Disease type	Course of disease	IgE (IU/mL)	IL-4 (pg/mL)	IL-6 (pg/mL)	IL-10 (pg/mL)	INF-*γ* (pg/mL)	CD3 (%positivity)	CD4 (%positivity)	CD8 (%positivity)
MPP (*n* = 680)	Acute phase	118.30 ± 29.41^∗▲^	3.12 ± 0.53^∗▲^	21.85 ± 4.01^∗▲^	10.73 ± 1.52^∗▲^	10.07 ± 3.18	62.90 ± 8.94	35.55 ± 8.20	22.57 ± 5.86
	Recovery phase	60.27 ± 30.01	2.22 ± 0.51	9.97 ± 1.41	4.68 ± 1.14	11.15 ± 3.73	57.24 ± 11.84	33.06 ± 10.17	19.97 ± 7.20
Bacterial pneumonia (*n* = 250)	Acute phase	22.65 ± 5.23	2.50 ± 0.41	120.11 ± 21.45	25.52 ± 2.01	8.64 ± 2.17	62.07 ± 7.86	29.76 ± 5.60	25.57 ± 9.01
	Recovery phase	22.42 ± 5.21	1.50 ± 0.50	23.21 ± 2.34	4.56 ± 1.01	8.75 ± 1.98	54.65 ± 6.21	29.45 ± 6.41	20.11 ± 8.32
Viral pneumonia (*n* = 400)	Acute phase	35.7 ± 7.89	2.70 ± 0.42	34.50 ± 5.17	14.50 ± 1.64	13.5 ± 2.50	52.40 ± 6.02	33.11 ± 5.67	17.86 ± 5.27
	Recovery phase	26.34 ± 10.64	1.62 ± 0.53	10.23 ± 2.16	3.26 ± 1.31	9.09 ± 1.87	52.87 ± 6.27	32.45 ± 7.91	18.01 ± 4.82
Normal control (*n* = 50)		19.93 ± 35.62	1.45 ± 0.40	6.00 ± 0.71	3.04 ± 1.20	8.83 ± 2.03	53.12 ± 5.95	29.10 ± 6.23	18.15 ± 4.62

*Compared with normal control group, *P* < 0.01. ^▲^Compared with recovery phase of *Mycoplasma pneumoniae* pneumonia, *P* < 0.01. Values represent mean ± SD.

## Abbreviations

MP: * Mycoplasma pneumoniae*
MPP: * Mycoplasma pneumoniae* pneumonia
ECP: Eosinophil Cationic Protein
IL: Interleukin
INF-*γ*: Interferon-*γ*
CPIS: Clinical pulmonary infection score.
